# Dickkopf-1 (Dkk-1) in plasma and synovial fluid is inversely correlated with radiographic severity of knee osteoarthritis patients

**DOI:** 10.1186/1471-2474-11-257

**Published:** 2010-11-10

**Authors:** Sittisak Honsawek, Aree Tanavalee, Pongsak Yuktanandana, Srihatach Ngarmukos, Natthaphon Saetan, Saran Tantavisut

**Affiliations:** 1 Department of Biochemistry, Faculty of Medicine, Chulalongkorn University, Bangkok 10330, Thailand; 2 Department of Orthopaedics, Faculty of Medicine, Chulalongkorn University, Bangkok 10330, Thailand

## Abstract

**Background:**

Osteoarthritis (OA) is a common degenerative joint disease causing pain, stiffness, reduced motion, swelling, crepitus, and disability. Dickkopf-1 (Dkk-1) is a critical mediator of osteoblastogenesis and regulates the joint remodeling. The aim of this study was to examine plasma and synovial fluid Dkk-1 levels of patients with primary knee OA and to investigate their relationship with disease severity.

**Methods:**

Thirty-five patients aged 55-83 years with knee OA and 15 healthy individuals were recruited into this study. Disease severity was determined using weight-bearing anteroposterior radiographs of the affected knee. The radiological grading of OA in the knee was performed according to the Kellgren-Lawrence grading system. Dkk-1 levels in both plasma and synovial fluid were evaluated using enzyme-linked immunosorbent assay.

**Results:**

The average concentration of circulating Dkk-1 in the knee OA patients was remarkably lower than that of healthy controls (396.0 ± 258.8, 95%CI 307.1-484.9 vs 2348.8 ± 2051.5, 95%CI 1164.3-3533.3 pg/ml, p < 0.0001). Dkk-1 levels in synovial fluid were significantly lower than in paired plasma samples (58.6 ± 31.8, 95%CI 47.7-69.6 vs 396.0 ± 258.8, 95%CI 307.1-484.9 pg/ml, p < 0.001). Furthermore, both plasma and synovial fluid Dkk-1 levels were inversely correlated with radiographic severity (r = -0.78, p < 0.001 and r = -0.42, p = 0.01, respectively). Plasma Dkk-1 levels were also significantly correlated with synovial fluid Dkk-1 levels (r = 0.72, p < 0.001).

**Conclusions:**

Dkk-1 levels in plasma and synovial fluid are inversely related to the severity of joint damage in knee OA. Dkk-1 could serve as a biochemical marker for determining disease severity and might play a potential role in the pathogenesis of the degenerative process of OA.

## Background

Osteoarthritis (OA) is the most prevalent joint disease causing pain, stiffness, reduced motion, swelling, crepitus, and disability. It is characterized by the progressive destruction of articular cartilage with joint-space narrowing, osteophyte formation, subchondral sclerosis, and synovitis [1]. The knee is the most clinically significant site of primary osteoarthritis involvement. One of the current methods to evaluate the affected joint is radiological assessment which reflects disease severity by grading the joint degeneration. The Kellgren-Lawrence grading scale representing disease severity has been the most widely used system [2]. The etiology and pathogenesis of OA remain poorly understood, but have been associated with several physiological factors such as obesity and aging [3]. Nevertheless, biochemical factors have by now been recognized as playing an important role in OA development.

Secreted glycoproteins of the Wingless (Wnt) signaling pathway are crucial regulators of cell growth and survival in a variety of human cell types. Wnt ligands bind to a receptor complex encompassing a member of the Frizzled family of seven transmembrane proteins and the co-receptor, low-density lipoprotein (LDL) receptor-related proteins (LRP5/6) [4]. In the canonical Wnt/β-catenin signaling pathway, receptor activation results in a stabilization of β-catenin, which accumulates and translocates into the nucleus to activate target gene expression. Dickkopf-1 (Dkk-1) is a secreted protein that has been defined as a direct inhibitor of Wnt/β-catenin signaling by interacting with the LRP5/6 co-receptors of frizzled [5,6]. Dkk-1 is a critical mediator of osteoblastogenesis and regulates the formation of the skeleton during the development of the embryo [7]. More recent studies have suggested a potential role of Dkk-1 in malignant bone disease and arthritis [8-10]. Uderhardt et al. have shown that inhibition of Dkk-1 effectively reduces bone erosion of sacroiliac joints [11]. In addition, Diarra and colleagues have documented that blockade of Dkk-1 reverses the bone-destructive pattern in a mouse model of rheumatoid arthritis to the bone-forming pattern of OA [10], indicating that Dkk-1 is a central regulator of joint remodeling. Recently, elevated circulating Dkk-1 levels have been associated with delayed progression of radiographic hip OA in women [12]. Furthermore, growing evidence has proposed an association between deregulated Wnt signaling components and joint disorders in OA cartilage chondrocyte cultures [13].

Even though circulating and/or synovial fluid levels of several cytokines have been investigated in patients with knee OA, there have not been any reports on the association of circulating and synovial fluid levels of Dkk-1 with disease activity in primary knee OA [14-18]. We have hypothesized that Dkk-1 in plasma and synovial fluid might be associated with the severity of clinical outcomes in knee OA patients. To prove this hypothesis, we have investigated the plasma and synovial fluid levels of Dkk-1 in knee OA patients and healthy controls. The aim of the present study was to evaluate, for the first time in the literature, both plasma and synovial fluid levels of Dkk-1 in patients with primary knee OA, and examine the possible relationships between plasma and synovial fluid Dkk-1 with the radiographic grading of knee osteoarthritis.

## Methods

### Study participants

This study was approved by the Institutional Review Board on Human Research of the Faculty of Medicine, Chulalongkorn University and was conducted in agreement with the Declaration of Helsinki. Written informed consent was obtained from the patients and healthy volunteers prior to their participation in this study.

Thirty-five patients aged 55 to 83 years with primary knee osteoarthritis (26 females and 9 males; mean age 68.8 ± 8.2 years) according to the criteria of the American College of Rheumatology were enrolled in the study. The severity of the disease was determined using weight-bearing anteroposterior radiographs of the affected knee. Knee radiographs were evaluated according to the Kellgren and Lawrence classification [2]: grade 1, doubtful narrowing of joint space and possible osteophytic lipping; grade 2, definite osteophytes and possible narrowing of joint space; grade 3, moderate multiple osteophytes, definite narrowing of joint space, some sclerosis and possible deformity of bone contour; grade 4, large osteophytes, marked narrowing of joint space, severe sclerosis and definite deformity of bone contour. The grading scale used for analysis was the one found higher upon comparison between both knees. We also recruited 15 gender and age matched subjects (10 females and 5 males; mean age 67.5 ± 4.6 years) with normal knee radiographs as controls. None of the participants had underlying diseases such as diabetes, histories of corticosteroid medication, other forms of arthritis, cancer, or other chronic inflammatory diseases.

### Laboratory methods

Synovial fluid was aspirated from the affected knee using sterile knee puncture just prior to surgery, when a total knee replacement was performed, centrifuged to remove cells and joint debris and stored immediately at -80°C until the day of measurement. No synovial fluid was extracted from the controls due to ethical concerns. Venous blood samples collected from the same patients on the day of surgery were centrifuged and stored at -80°C until utilized. Double-blind quantitative detection of Dkk-1 in plasma and synovial fluid was performed by sandwich enzyme-linked immunosorbent assay (ELISA) using a commercially available test kit according to the manufacturer's protocol (Quantikine, R&D Systems, Minneapolis, MN). Briefly, standards of recombinant human Dkk-1, plasma, and synovial fluid samples prediluted 1:4 in assay buffer were added to 96-well microtiter plates precoated with mouse monoclonal antibody against Dkk-1 and incubated for 2 hours at room temperature. The wells were then washed four times with washing buffer and incubated for 2 hours at room temperature with a horseradish peroxidase-conjugated goat polyclonal antibody against Dkk-1. After four washes, substrate solution was added to each well, and the plate was incubated for 30 minutes at room temperature in the dark. Finally, the reaction was stopped with the stop solution, and absorbance was measured at 450 nm using an automated microplate reader. Recombinant human Dkk-1 was used to generate a linear standard calibration curve (range 31.2-2,000 pg/ml). The manufacturer-reported precision was 3.3-4.2% (intra-assay) and 4.6-7.6% (inter-assay). The sensitivity of this assay was 4.2 pg/ml.

### Statistical analysis

Statistical analysis was carried out using the statistical package for social sciences (SPSS) software, version 16.0 for Windows. Tests of normality and test of homogeneity of variances were performed to determine the subject's age, body mass index (BMI) and Dkk-1 concentration in the plasma and synovial fluid. The analysis of co-variance (ANCOVA) indicated that age, gender and BMI were not potentially confounding factors in the study. Demographic data between patients and controls were compared by Chi-square tests and unpaired Student's *t *tests, where appropriate. Comparisons between the groups were performed using one-way analysis of variance (ANOVA) with Tukey post hoc test if ANOVA showed significance. Comparisons between groups were made using Mann-Whitney *U *test (for two groups) or Kruskal-Wallis test (for more than two groups) when the variances were not equal among the groups. Pearson's correlation coefficient was employed to determine the correlation among the concentration of Dkk-1 in the plasma and synovial fluid and the disease severity. Sensitivity, specificity, receiver-operating characteristic (ROC) curves were also determined. P values < 0.05 were considered to be statistically significant for differences and correlations. All values are expressed as mean ± standard deviation (SD) and 95% confidence intervals (95%CI).

## Results

**Figure 1:**
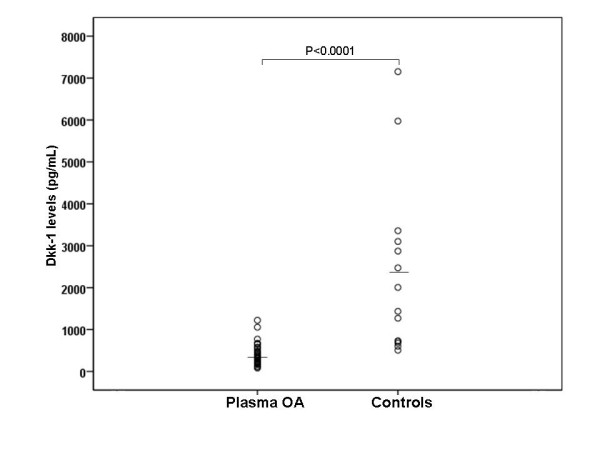
**Plasma Dkk-1 levels of patients with osteoarthritis (n = 35) and healthy controls (n = 15)**.

Thirty-five plasma and synovial fluid samples from knee OA patients and 15 plasma samples from healthy controls were acquired for measurement of Dkk-1 concentrations. Characteristics of the study population are shown in Table 1. There was no clinically meaningful difference in age between OA patients and controls (68.8 ± 8.2, 95%CI 66.3-70.5 vs 67.5 ± 4.6, 95%CI 65.3-70.3 years, p = 0.6). In addition, the female/male ratio was 26/9 in patients and 10/5 in controls (p = 0.1). The study population was adjusted for age and gender. There was no significant difference in body mass index between OA patients and controls (26.6 ± 3.8, 95%CI 25.3-28.0 vs 25.5 ± 1.3, 95%CI 24.6-26.4 kg/m^2^, p = 0.3). As demonstrated in Figure 1, OA patients had lower plasma Dkk-1 concentrations compared to healthy controls (396.0 ± 258.8, 95%CI 307.1-484.9 vs 2348.8 ± 2051.5, 95%CI 1164.3-3533.3 pg/ml, p < 0.0001). Dkk-1 levels in synovial fluid were significantly lower than in paired plasma samples (58.6 ± 31.8, 95%CI 47.7-69.6 vs 396.0 ± 258.8, 95%CI 307.1-484.9 pg/ml, p < 0.001). In the OA group, there were no differences in Dkk-1 levels between males and famales in either plasma (400.7 ± 275.1, 95%CI 291.9-509.5 vs 380.1 ± 209.1, 95%CI 205.3-555.0 pg/ml, p = 0.8) or synovial fluid (72.1 ± 60.2, 95%CI 21.8-122.5 vs 54.6 ± 16.6, 95%CI 48.1-61.2 pg/ml, p = 0.5). In the control group, plasma Dkk-1 concentrations were not significantly different between both genders (1831.7 ± 1174.7, 95%CI 373.1-3290.3 vs 2636.1 ± 2426.8, 95%CI 770.6-4501.5 pg/ml, p = 0.4).

**Table 1:** Characteristics of knee osteoarthritis patients and controls.

	Controls (95%CI)	OA patients (95%CI)			
		Total	KL grage 2	KL grade 3	KL grade 4
N	15	35	10	12	13
Age (years)	67.5 ± 4.6	68.8 ± 8.2	68.8 ± 6.8	68.1 ± 8.3	69.5 ± 7.5
	(65.3-70.3)	(66.3-70.5)	(63.9-73.6)	(61.5-74.6)	(64.9-73.9)
Female/Male	10/5	26/9	7/3	9/3	10/3
BMI (kg/m^2^)	25.5 ± 1.3	26.6 ± 3.8	26.2 ± 1.4	26.4 ± 3.1	27.2 ± 4.8
	(24.6-26.4)	(25.3-28.0)	(25.2-27.2)	(24.5-28.4)	(23.7-30.7)

Data are expressed as mean and SD.

BMI = Body mass index.

KL = Kellgren and Lawrence

**Figure 2:**
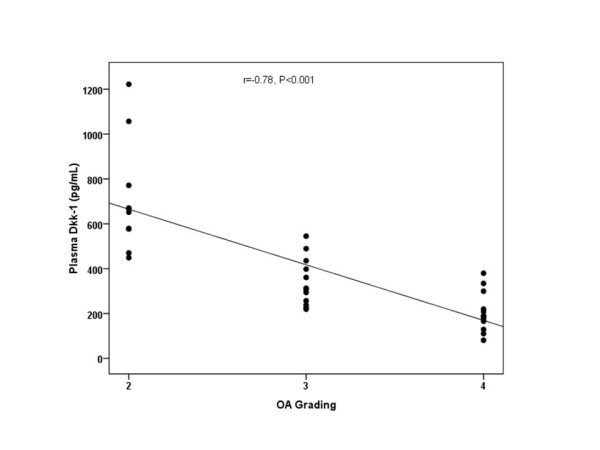
**Scattergram showing the inverse correlation between plasma Dkk-1 levels in patients with OA and severity classified according to Kellgren and Lawrence grading scale (r = -0.78, p < 0.001)**.

**Figure 3:**
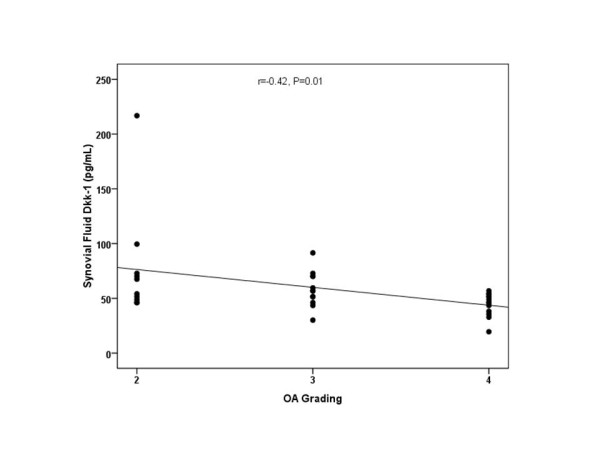
**Scattergram showing the negative correlation between synovial fluid Dkk-1 levels in patients with OA and severity classified according to Kellgren and Lawrence grading scale (r = -0.42, p = 0.01)**.

**Figure 4:**
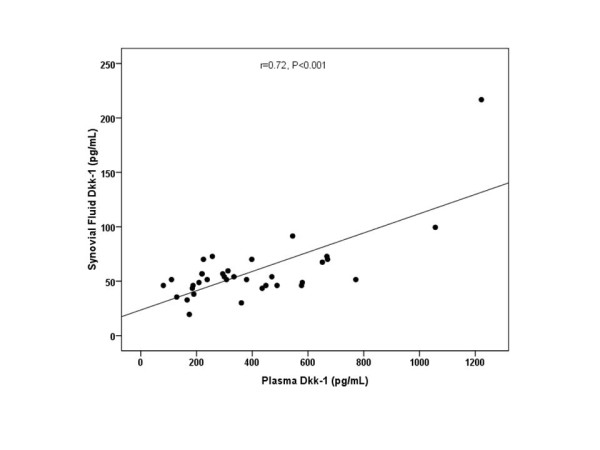
**Scattergram showing the positive correlation between plasma and synovial fluid Dkk-1 concentrations in OA patients (r = 0.72, p < 0.001)**.

According to the Kellgren and Lawrence (KL) classification, 10 patients were KL grade 2, whereas 12 patients were KL grade 3, and 13 patients were KL grade 4 osteoarthritis. The circulating and synovial fluid levels of Dkk-1 were assessed and compared in association with radiological KL grading of OA. The plasma Dkk-1 levels from KL grade 2 were 711.4 ± 248.0, 95%CI 534.0-888.9 pg/ml; those from KL grade 3 were 340.1 ± 107.0, 95%CI 272.1-408.1 pg/ml; and those from KL grade 4 were 204.9 ± 86.6, 95%CI 152.6-257.2 pg/ml (Figure 2). These results showed that plasma Dkk-1 levels in KL grade 3 and 4 were significantly lower than those of KL grade 2 (p < 0.001). Although the mean plasma levels of Dkk-1 in KL grade 4 were lower than those in KL grade 3, the difference was not significant (p = 0.1). Furthermore, the synovial fluid levels of Dkk-1 from KL grade 2 were 77.3 ± 51.7, 95%CI 40.3-114.3 pg/ml; those from KL grade 3 were 58.4 ± 16.1, 95%CI 48.1-68.6 pg/ml; and those from KL grade 4 were 44.5 ± 10.6, 95%CI 38.1-50.9 pg/ml (Figure 3). The data showed that synovial fluid Dkk-1 levels in KL grade 2 and 3 were significantly elevated compared with those of KL grade 4 (p = 0.04). Although synovial fluid Dkk-1 levels in KL grade 2 were elevated compared with those of KL grade 3, the difference was not significant (p = 0.4). We further analyzed the correlation between the plasma and synovial fluid levels of Dkk-1 and the severity of osteoarthritis. Intriguingly, plasma Dkk-1 levels were negatively correlated with the radiographic grading of knee OA (r = -0.78, p < 0.001) (Figure 2). Synovial fluid levels of Dkk-1 were weakly associated with the radiographic severity of disease (r = -0.42, p = 0.01) (Figure 3). Figure 4 reveals that a rough estimate of plasma Dkk-1 levels in OA patients is 200-400 pg/ml whereas that of synovial fluid Dkk-1 levels is 25-70 pg/ml. However, plasma Dkk-1 levels showed a positive correlation with synovial fluid Dkk-1 levels (r = 0.72, p < 0.001). Using ROC curves drawn with data of our results, the cutoff value was set to provide optimal diagnostic accuracy and likelihood ratios for Dkk-1 protein. A plasma level of 459.5 pg/ml was with a sensitivity of 90% and a specificity of 92% for moderate knee OA (KL grade 3). A plasma level of 303.5 pg/ml was with a sensitivity of 77% and a specificity of 85% for advanced knee OA (KL grade 4).

## Discussion

The Wnt signaling pathway plays an essential role in cell patterning, proliferation, differentiation, and fate determination during embryogenesis and therefore it is not surprising that Wnt modulators, including Dkks are also involved. Dkk is a family of cysteine-rich proteins consisting of Dkk-1, Dkk-2, Dkk-3, Dkk-4 and a unique Dkk-3-related protein "soggy" [19]. Dkk-1 serves as a natural antagonist of the Wnt signaling pathway and plays substantial roles in vertebrate embryogenesis including head induction, skeletal development, and limb patterning [20,21]. Deletion of a single allele of Dkk-1 enhances bone mass in mice [22]. A recent study has demonstrated that aberrant expression of Dkk-1 in myeloma cells was associated with increased bone erosion in human multiple myeloma [23]. Therefore, expression of Dkk-1 in inflammatory and degenerative joint diseases may block bone formation within the joint.

It has been previously demonstrated that circulating Dkk-1 is present in rheumatoid arthritis, ankylosing spondylitis, and osteoarthritis [24-26]. However, the association between circulating and synovial fluid levels of Dkk-1 and disease severity has never been specifically evaluated in knee OA patients. To our knowledge, data on the relationship between Dkk-1 levels in plasma and synovial fluid and severity of knee OA have as yet not been reported in the literature. This study has been the first to illustrate that Dkk-1 was detected in both plasma and synovial fluid derived from patients with primary knee OA, and that Dkk-1 were inversely related to radiographic grading of knee OA.

The most intriguing finding in this study has been that concentrations of Dkk-1 were decreased in plasma of patients with primary knee OA compared to the controls. Our results suggest that there is reduced systemic production of Dkk-1 in knee OA. It should be noted that Dkk-1 levels in synovial fluid were significantly lower than those seen in paired plasma samples. The source of Dkk-1 could be derived from the local tissues (inflamed synovium, cartilage, and subchondral bone) and extra-articular tissues. Previous studies have shown that Dkk-1 was expressed in synovial cells, articular cartilage chondrocytes and subchondral bone osteoblasts in OA knees [10,27,28]. Dkk-1 levels in plasma and synovial fluid were measured in a well-defined knee OA population at every stage of disease, and were significantly lower in end-stage knee OA patients compared with early OA patients. This observation suggests a significant reduction in the systemic and local expression of Dkk-1 in patient with advanced knee OA. The mechanisms of Dkk-1 reduction in the circulation and synovial fluid of OA patients remain to be investigated further.

In concordance with our findings, Voorzanger-Rousselot and coworkers have revealed that circulating Dkk-1 levels were lower in patients with knee OA compared with healthy controls [26]. In our study circulating and synovial fluid levels of Dkk-1 were higher in early knee OA patients (KL grade 2) compared to end-stage knee OA (KL grade 4), in agreement with Lane's observation. Recently, Lane and colleagues have documented that increased circulating levels of Dkk-1 appeared to be associated with delayed progression of radiographic hip OA in elderly women [12]. Dkk-1 has been demonstrated to delay new bone formation and subchondral bone remodeling and is a potent negative regulator of osteoblast differentiation [22,23]. Therefore, the mild/moderate knee OA patients with high circulating and synovial fluid levels of Dkk-1 may reflect the Dkk-1 capability to inhibit bone remodeling around the osteoarthritic joint. It is interesting to postulate that Dkk-1 might be able to delay articular cartilage loss. In this study, patients with advanced articular cartilage loss were found to be the subgroup with the lowest circulating and synovial fluid levels of Dkk-1. Previous reports have demonstrated that increased Dkk-1 levels were correlated with the pathogenesis of joint disorders [10,26,27]. In this study, Dkk-1 levels were decreased in direct relation to the severity of knee OA. Moreover, plasma Dkk-1 in OA patients was remarkably lower than in controls. These conflicting results may be attributable to differences in disease advancement, populations or assays applied, or to incomplete control of confounding variables.

It is interesting to point out that in a mouse model of osteoarthritis [27] and in human OA patients [28,29] the loss of a souble Wnt antagonist, frizzled-related protein-3, was associated with enhanced cartilage damage, plausibly by modulating the activity of chondrocytes [30]. This may provide an explanation for the inverse correlation between Dkk-1 and disease severity in primary knee OA patients. Furthermore, decreased Dkk-1 levels tend to be essential for osteophyte formation, suggesting that Wnt signaling is a critical initiator for bone formation in the joint. Local bone formation in the presence of osteophyte formation is a hallmark of degenerative joint diseases including osteoarthritis [10]. Additional studies will be required to determine whether the concentrations of Dkk-1 in synovial fluid and plasma are related to the expression of Dkk-1 in joint tissues. The mechanisms underlying Dkk-1 reduction in the circulation and synovial fluid of OA patients remain to be further investigated.

In this study, we have been aware of several potential limitations. First, our study was based on a small sample size of enrolled patients. A further study conducted on a random sample of a larger population will be needed to substantiate our results. Secondly, only Dkk-1 concentration has been measured in both plasma and synovial fluid. Additional immunohistochemical investigations of Dkk-1 expression could provide further valuable information on the pathogenic role of Dkk-1 in OA. Thirdly, we have not investigated the role of other soluble Wnt inhibitors, such as frizzled-related protein-3, which has been proposed to be associated with joint destruction in OA [31,32]. Moreover, the daily variation of plasma Dkk-1 has yet to be fully determined and it is unclear which physical activities might influence plasma levels (walking, jumping, eating, etc.). Therefore, diurnal and activity-related variations in plasma Dkk-1 will be needed to be further evaluated. Finally, as this study has been designed as a cross-sectional study it is not possible to determine a definite cause and effect relationship and to arrive at a strong conclusion. However, prospective longitudinal studies are warranted to demonstrate disease progression and define the exact role of Dkk-1 in knee OA.

## Conclusions

This study has revealed a significant decrease in plasma Dkk-1 of OA patients and illustrated a pronounced inverse correlation with the degree of radiographic severity in patients with primary knee OA. The data of this study support the finding that Dkk-1 may be a useful prognostic parameter to reflect the disease severity of primary knee OA. In addition, Dkk-1 levels in plasma were directly correlated with those in synovial fluid. This study has been the first to investigate such a correlation and further studies will be required to define the mechanisms underlying this association. Additional investigations will be required to shed light on the possible role of Dkk-1 involved in the pathogenesis of chronic degenerative joint disorder, with the aim of developing effective pharmacological agents to delay the progression to osteoarthritis.

## Abbreviations

ANCOVA: analysis of co-variance; ANOVA: analysis of variance; BMI: body mass index; CI: confidence intervals; DKK-1: Dickkopf-1; ELISA: enzyme-linked immunosorbent assay; KL: Kellgren and Lawrence; LDL: low-density lipoprotein; LRP: low-density lipoprotein receptor-related proteins; OA: osteoarthritis; ROC: receiver-operating characteristic; SD: standard deviation; SPSS: statistical package for social sciences; WNT: Wingless.

## Competing interests

The authors declare that they have no competing interests.

## Authors' contributions

SH was responsible for the conception and design of the study, the analysis and interpretation of the data, and drafting of the manuscript. AT, PY, and SN performed operations, acquired patients, and contributed to the sample collection. NS and ST assisted sample collection and data analysis. All authors have read and approved the final manuscript.

## Pre-publication history

The pre-publication history for this paper can be accessed here:

http://www.biomedcentral.com/1471-2474/11/257/prepub
